# Purification and biochemical properties of a cytochrome *bc *complex from the aerobic hyperthermophilic archaeon *Aeropyrum pernix*

**DOI:** 10.1186/1471-2180-11-52

**Published:** 2011-03-14

**Authors:** Yoshiki Kabashima, Junshi Sakamoto

**Affiliations:** 1 Department of Bioscience and Bioinformatics, Kyushu Institute of Technology, Kawazu 680-4, Iizuka, Fukuoka-ken 820-8502, Japan; 2 Department of Chemistry, School of Medicine, Kyorin University, Mitaka, Tokyo 181-8611, Japan

## Abstract

**Background:**

The bioenergetics of Archaea with respect to the evolution of electron transfer systems is very interesting. In contrast to terminal oxidases, a canonical *bc*_1 _complex has not yet been isolated from Archaea. In particular, *c*-type cytochromes have been reported only for a limited number of species.

**Results:**

Here, we isolated a *c*-type cytochrome-containing enzyme complex from the membranes of the hyperthermophilic archaeon, *Aeropyrum pernix*, grown aerobically. The redox spectrum of the isolated *c*-type cytochrome showed a characteristic α-band peak at 553 nm corresponding to heme C. The pyridine hemochrome spectrum also revealed the presence of heme B. In non-denaturing polyacrylamide gel electrophoresis, the cytochrome migrated as a single band with an apparent molecular mass of 80 kDa, and successive SDS-PAGE separated the 80-kDa band into 3 polypeptides with apparent molecular masses of 40, 30, and 25 kDa. The results of mass spectrometry indicated that the 25-kDa band corresponded to the hypothetical cytochrome *c *subunit encoded by the ORF *APE_1719.1*. In addition, the *c*-type cytochrome-containing polypeptide complex exhibited menaquinone: yeast cytochrome *c *oxidoreductase activities.

**Conclusion:**

In conclusion, we showed that *A. pernix*, a hyperthemophilic archaeon, has a "full" *bc *complex that includes a *c*-type cytochrome, and to the best of our knowledge, *A. pernix *is the first archaea from which such a *bc *complex has been identified. However, an electron donor candidates for cytochrome *c *oxidase, such as a blue copper protein, have not yet been identified in the whole genome data of this archaeon. We are currently trying to identify an authentic substrate between a *bc *complex and terminal oxidase.

## Background

*Aeropyrum pernix *is a hyperthermophilic crenarchaeon isolated from the seas of Japan, and its complete genome sequence has been reported [1,2]. Most of the hyperthermophilic archaea grow anaerobically, but this archaeon is strictly aerobic and grows optimally at 90-95°C at neutral pH. Analysis of the respiratory chain of the organism is important for understanding the mechanism of aerobic growth in such environments. However, there are only a few reports about the bioenergetics of *A. pernix*.

**Figure 1:**
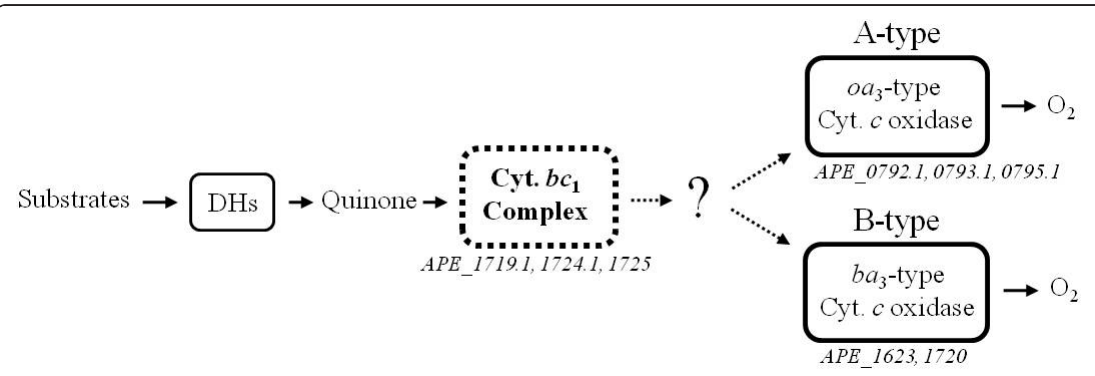
**Schematic representation of the respiratory chain of *Aeropyrum pernix *K1**. Genes encoding cytochrome *c *oxidase and other respiratory components in the bacterium are indicated. ORFs *APE_1719.1*, *APE_1724.1 *and *APE_1725 *encode the cytochrome *c*_553 _complex which was isolated in this study. ORFs *APE_0792.1*, *APE_0793.1 *and *APE_0795.1*, annotated as *aoxABC *genes, encode an A-type cytochrome *c *oxidase, and ORFs *APE_1623 *and *APE_1720 *encode a B-type cytochrome *c *oxidase. In the previous study of Ishikawa *et al*. (2002), these 2 terminal oxidases were designated as cytochrome *aa*_3_- and *ba*_3_-type cytochrome *c *oxidase, respectively.

Many bacteria and archaea have 2 to 6 terminal oxidases in the respiratory chain [3]. The heme-copper oxidase superfamily can be classified into 3 subfamilies (A-, B-, and C-type) on the basis of the amino acid sequence of subunit I [4,5]. The group of A-type oxidases includes mitochondrial cytochrome *aa*_3_-type cytochrome *c *oxidase (complex IV) and many other bacterial oxidases. In contrast, B-type oxidases have been identified mainly from extremophiles, including thermophilic bacteria, such as *Geobacillus thermodenitrificans *(formerly called *Bacillus thermodenitrificans*) [6,7] and *Thermus thermophilus *[8], and archaea, such as *Sulfolobus acidocaldarius *[9]. Analysis of the complete genome sequence of *A. pernix *has shown that it contains A- and B-type heme-copper terminal oxidases (Figure 1). Ishikawa *et al*. isolated 2 terminal oxidases from *A. pernix *and designated them as cytochrome *ba*_3_-type (B-type) and *aa*_3_-type (A-type) cytochrome *c *oxidases, respectively [10]. Both oxidases have a Cu_A _binding motif, but its substrates have not been identified in the genome sequence.

An extremely haloalkaliphilic archaeon, *Natronomonas pharaonis*, uses a blue copper protein named halocyanin as a substrate for the terminal oxidase instead of cytochrome *c *[11]. In *S. acidocaldarius*, a blue copper protein named sulfocyanin, which is a part of the SoxM supercomplex, is an intermediate in the electron transfer from the *bc*_1_-analogous complex to the terminal oxidase [12]. However, no genes for blue copper proteins homologous to halocyanin or sulfocyanin have been found in the genome of *A. pernix*. Therefore, although these oxidases can use *N*,*N*,*N'*,*N*'-tetramethyl-*p*-phenylenediamine (TMPD) and/or bovine cytochrome *c *as substrates *in vitro*, the authentic substrate of the two terminal oxidases is not known.

In contrast to terminal oxidases, complex III of archaea is not well-known and a canonical *bc*_1 _complex has not been identified in any archaeal genome [13]. Among the subunit components of the *bc *complex, both cytochrome *b *and Rieske Fe/S protein are widely conserved, while the *c*-type cytochrome subunit has diverged into several classes, some of which show no sequence similarity to the class of cytochrome *c*_1 _subunits [14]. This is compatible with the view that the functional and evolutionary core of the *bc *complex includes cytochrome *b *and the peripheral domain of the Rieske Fe/S protein and that different *c*-type cytochromes have been recruited independently several times during molecular evolution. Generally, a *c*-type cytochrome has been reported only for a limited number of archaeal species, such as halophiles and thermoacidophiles, in contrast to *a/o*-type and *b*-type cytochromes, which seem ubiquitous in the respiratory chains of archaeal species. Focusing on homologues of the cytochrome *bc *components, cytochrome *b *and Rieske Fe/S proteins are present in some archaeal species, such as *Sulfolobus*, and constitute supercomplexes with oxidase subunits [15], whereas cytochrome *c *components are missing even in those organisms. Several *bc*_1_-analogous complexes have been identified thus far in archaea such as *Halobacterium salinarum *[16] and *Acidianus ambivalens *[17].

In this study, we isolated *c*-type cytochromes from the membranes of *A. pernix *K1 cells and characterized the spectroscopic and enzymatic properties of the cytochromes. Our data indicate that the isolated *c*-type cytochrome is equivalent to the cytochrome *c *subunit of the *bc *complex and forms a supercomplex with cytochrome *c *oxidase.

## Results

### Isolation of a membrane bound *c*-type cytochrome from *A. pernix*

We isolated a membrane bound *c*-type cytochrome from the membranes and designated it cytochrome *c*_553_. A cytochrome oxidase was also isolated and designated cytochrome *oa*_3 _oxidase, as shown later. *A. pernix *K1 cells were harvested in the early stationary phase, and membranes were prepared. The membrane proteins were solubilized with DDM and fractionated using 3-step chromatography. In the first DEAE-Toyopearl column chromatography, the cytochrome *c*_553 _and cytochrome *oa*_3 _oxidase were mainly eluted with 100 mM NaCl (data not shown). Also in the second Q-Sepharose column chromatography, the cytohrome *c*_553 _eluted together with the cytochrome *oa*_3 _oxidase at ~200 mM NaCl (Additional file 1). Interestingly, the peak fractions from Q-Sepharose, including cytochrome *c*_553 _and *oa*_3 _oxidase, showed not only TMPD oxidation activity (4.1 μmol min^-1^mg^-1^) but also menaquinol oxidation activity (1.0 μmol min^-1^mg^-1^). This suggested that cytochrome *c*553 and cytochrome *c *oxidase interact. Subsequent chromatography on a hydroxyapatite column separated the cytochrome *c*_553 _and cytochrome *oa*_3 _oxidase into 2 peaks (Additional file 2). Table 1 shows a summary of the purification of cytochrome *c*_553_. The *c*-type cytochrome content was enriched approximately 9.6-fold during the purification.

**Table 1:** Purification of *A. pernix *cytochrome *c*_553_.

Steps	Total Protein (mg)	*c*-type cytochrome	
		
		Total (nmol)	Specific (nmol mg^-1^)
Membranes	589	463	0.787
DDM extract	494	344	0.697
DEAE-Toyopearl	27.1	105	3.88
Q-Sepharose	21.1	30.8	1.46
Hydroxyapatite	2.00	15.1	7.55

### Spectroscopic properties of cytochromes in *A. pernix*

**Figure 2:**
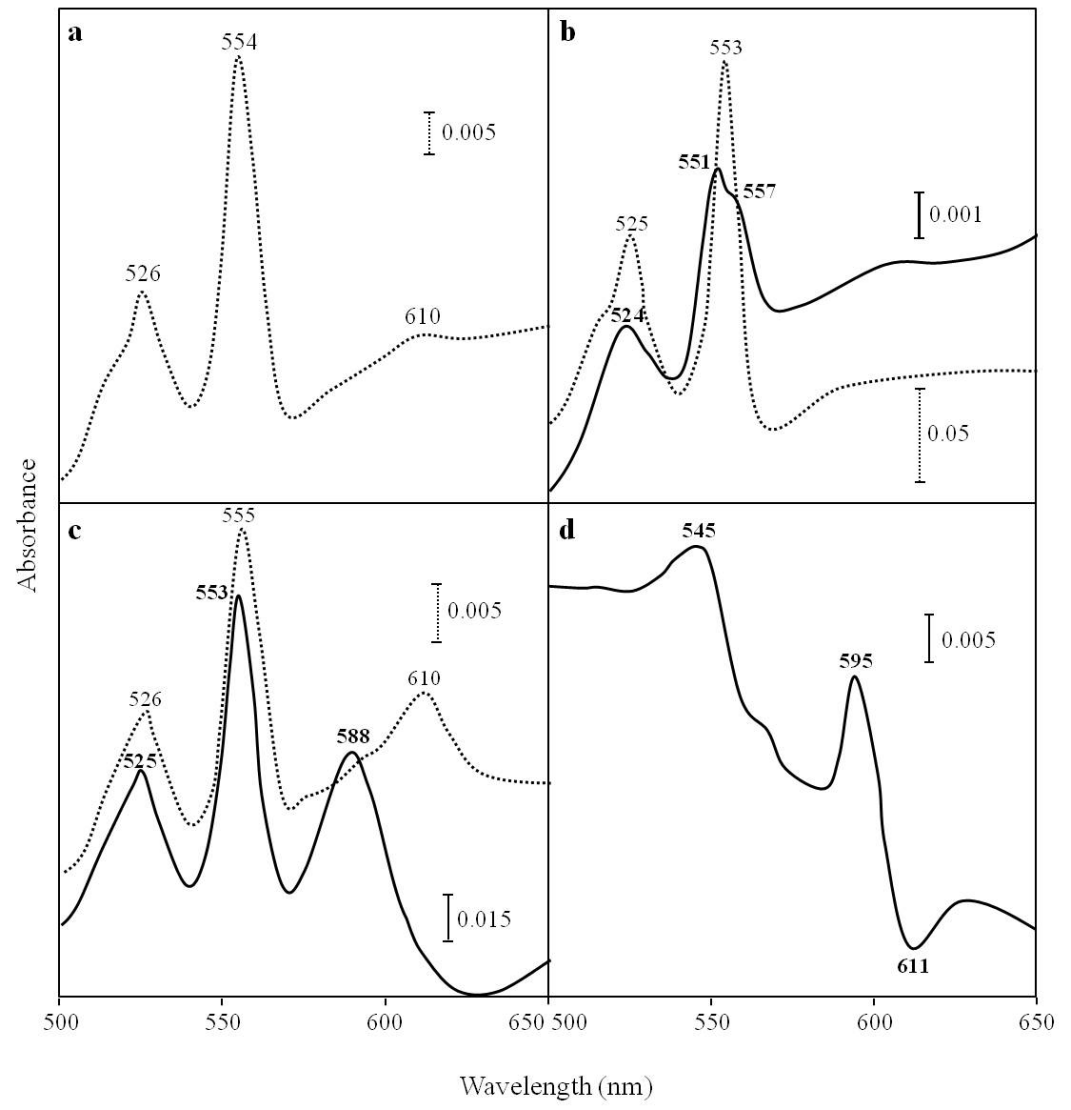
**Spectra of cytochromes in *A. pernix***. Difference spectrum in the sodium dithionite-reduced form *minus *the air-oxidized form (*dotted line*) and pyridine ferro-hemochromes (*solid line*) of membranes (**a**), cytochrome *c*_553 _(**b**), and cytochrome *oa*_3 _oxidase (**c**). To measure a spectrum of membranes, they were solubilized with 5% (w/v) Triton X-100, as described in Materials and Methods. Difference spectrum of the CO-reduced *minus *the reduced forms of cytochrome *oa*_3 _oxidase (**d**). The partially purified oxidase was reduced with sodium dithionite (baseline) and then bubbled with CO gas for 1 min.

**Figure 3:**
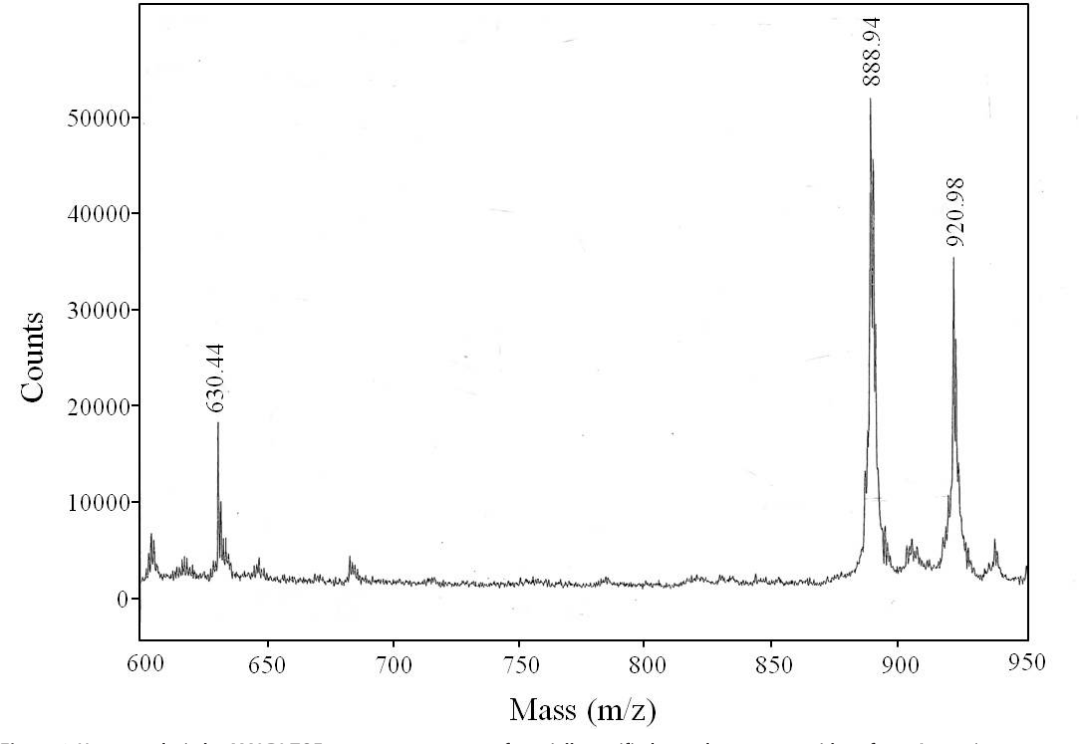
**Heme analysis by MALDI-TOF mass spectrometry of partially purified cytochrome *oa*_3 _oxidase from *A. pernix***. Heme was extracted from the oxidase preparation by shaking vigorously with acetone-HCl, followed by extraction with ethyl acetate. The extracted heme was analyzed by MALDI-TOF mass spectrometry as detailed in the "Materials and Methods".

The redox difference spectrum of membranes showed α-band peaks with maxima at 554 and 610 nm (Figure 2a), derived from *c*- and *a*-type cytochromes, respectively. The isolated cytochrome *c*_553 _in the reduced state showed an absorption peak at 553 nm (Figure 2b, *dotted line*). The pyridine ferro-hemochrome spectrum showed 2 α-band peaks with maxima at 551 and 557 nm, indicating the presence of heme C and heme B (Figure 2b, *solid line*) [18]. The redox spectrum of the cytochrome *oa*_3 _oxidase showed α-band peaks with maxima at 555 and 610 nm (Figure 2c, *dotted line*) and the pyridine ferro-hemochrome spectrum did α-band peaks with maxima at 553 and 588 nm (Figure 2c, *solid line*), indicating the presence of heme O and heme A [18,19]. To determine the heme species of the oxidase in more detail, total heme was extracted from the partially purified oxidase preparation and analyzed by mass spectrometry. We observed 3 peaks at molecular masses of 630.44, 888.94, and 920.98 (Figure 3). The molecular mass of 888.94 matches that of heme O_p1_, which was identified in *Sulfolobus *and other species [20], while the molecular mass of 920.98 matches that of heme A_s_. The molecular mass of 630.44 matches that of heme B, which is probably contamination from other cytochromes, because the peak height is lower than those of hemes O_p1 _and A_s_, and this oxidase does not contain *b*-type heme (Figure 2c). The difference spectrum of the CO-bound, reduced form minus the reduced form showed a peak and a trough at 595 nm and 611 nm, respectively, in the α region (Figure 2d) and those at 432 nm and 444 nm in the γ region (data not shown), indicating that CO was bound to an *a*-type heme (Figure 2d), and thus the oxidase was designated a cytochrome *oa*_3_-type.

### Polypeptide composition and enzyme activities

**Figure 4:**
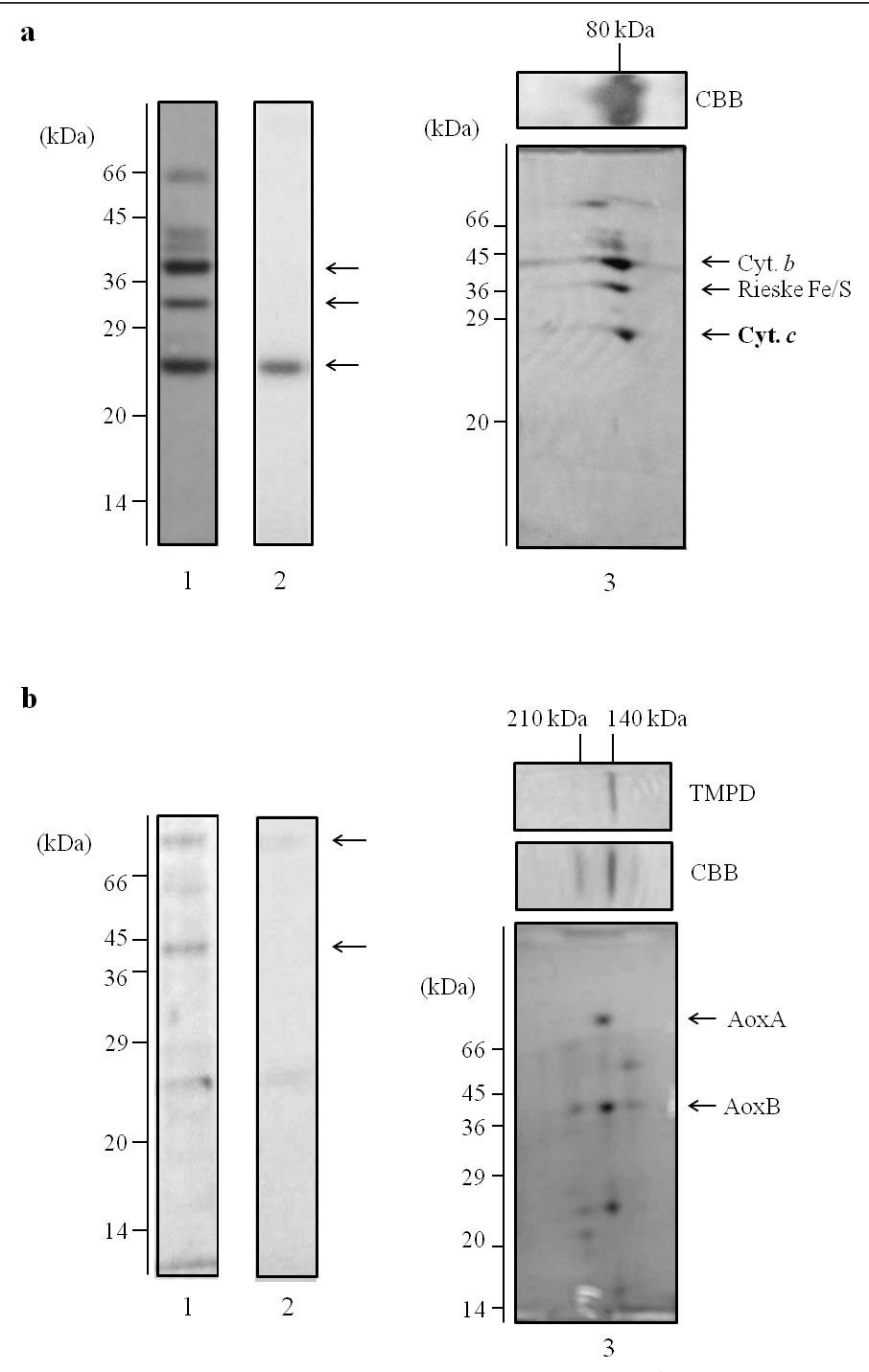
**SDS-PAGE (*panel 1 *and *2*) and Two-dimensional electrophoresis analysis (*panel 3*) of the cytochrome *c*_553 _(a) and cyothcrome *oa*_3 _oxidase (b) from *A. pernix***. The acrylamide concentration of the SDS-PAGE gel was 13.5%. The gel was stained for protein with CBB (*panel 1*) and for heme with *o*-toluidine in the presence of H_2_O_2 _(*panel 2*). The samples were analyzed by BN-PAGE (horizontal) and then SDS-PAGE (vertical, *panel 3*). A 5-18% acrylamide gradient gel was used for native PAGE, and the gels were stained with CBB. The cytochrome *oa*_3 _oxidase was revealed by its TMPD oxidation activity (**b ***panel 3*). The acrylamide concentration of the second dimension SDS-PAGE gel was 15%, and the gels were stained with CBB. Side bars indicate the molecular mass standards. The arrows indicate the corresponding subunits of the cytochrome *c*_553 _and cytochrome *oa*_3 _oxidase.

**Figure 5:**
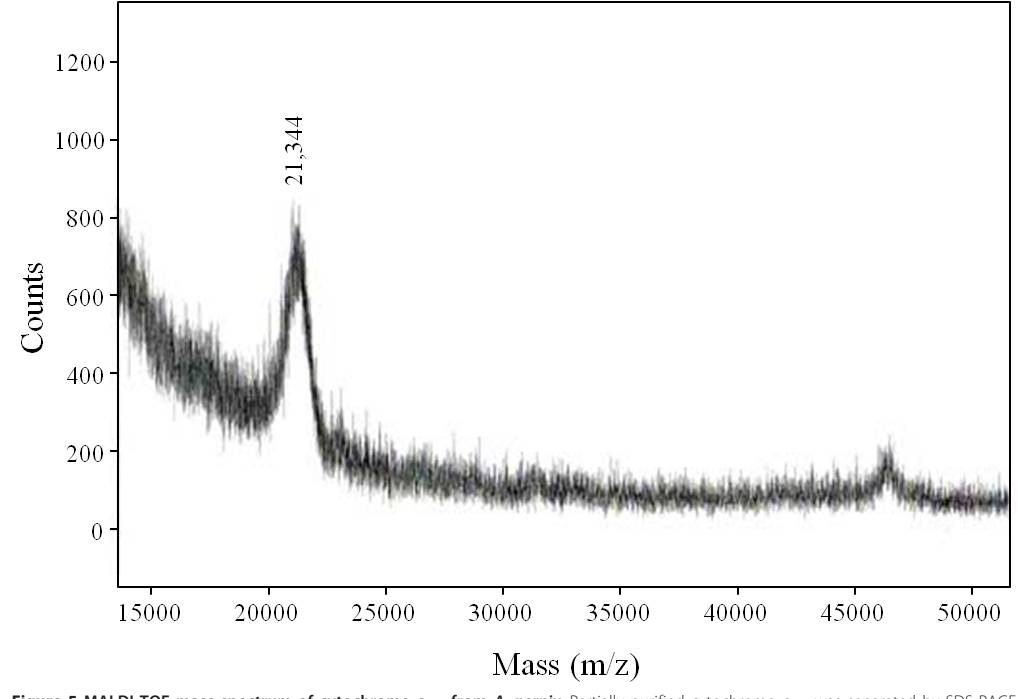
**MALDI-TOF mass spectrum of cytochrome *c*_553 _from *A. pernix***. Partially purified cytochrome *c*_553 _was separated by SDS-PAGE (Figure 4a, *panel 1*), and the 25-kDa band was extracted from the acrylamide gel. Mass spectrum analysis was performed as detailed in the Materials and Methods.

SDS-PAGE showed mainly 3 polypeptide bands for cytochrome *c*_553 _with apparent molecular masses of 40, 30, and 25 kDa (Figure 4a, *panel 1*). The 25-kDa band was visualized with heme staining (Figure 4a, *panel 2*). We performed mass analysis for the 3 bands at 40, 30, and 25 kDa using a MALDI-TOF/MS spectrometer. The 40- and 30-kDa polypeptides could not be identified. The 25-kDa polypeptide, which was positive for heme staining, had a molecular mass of 21,344 (Figure 5). The theoretical mass of the *APE_1719.1 *gene, which encodes the hypothetical cytochrome *c *subunit of the *bc *complex, was 20,813. The calculated mass of the *APE_1719.1 *gene product, which is the hypothetical cytochrome *c *polypeptide of the *bc *complex, is 21,429. On a BN-PAGE gel, cytochrome *c*_553 _migrated at 80 kDa as a single band (Figure 4a, *panel 3*). The entire panel was excised and processed by two-dimensional SDS-PAGE. The 80-kDa band consisted of 3 main polypeptides as shown by SDS-PAGE (Figure 4a, *panel 1 *and *panel 3*) indicating that these 3 polypeptides form a complex. For partially purified cytochrome *oa*_3 _oxidase, SDS-PAGE showed 3 polypeptide bands with apparent molecular masses of 74, 40, and 25 kDa (Figure 4b, *panel 1*). The 25-kDa band was visualized by heme staining, suggesting this band was derived from cytochrome *c*_553 _(Figure 4b, *panel 2*). BN-PAGE showed a band at 140 kDa, which had TMPD oxidase activity, suggesting that the band contain a cytochrome *c *oxidase (Figure 4b, *panel 3*). The 140-kDa band was separated by SDS-PAGE and found to consist of 3 main polypeptides as shown by SDS-PAGE (Figure 4b, *panel 1 *and *panel 3*).

The isolated cytochrome *oa*_3 _oxidase had TMPD and yeast cytochrome *c *oxidation activity, with values of 132 and 0.68 μmol min^-1 ^mg^-1^, respectively, while the cytochrome *c*_553 _complex did not show any oxidase activity. On the other hand, cytochrome *c*_553 _oxidized menaquinol and reduced yeast cytochrome *c *(3.7 μmol min^-1 ^mg^-1^), i.e. showed activity similar to that of quinone: cytochrome *c *oxidoreductase, while isolated cytochrome *oa*_3 _did not oxidize menaquinol. Interestingly, after adding the fractions containing cytochrome *c*_553 _to cytochrome *oa*_3 _oxidase, TMPD oxidase activity increased ~ 5.0-fold (132 μmol min^-1 ^mg^-1 ^vs 664 μmol min^-1 ^mg^-1^).

## Discussion

In this study, we isolated a membrane bound cytochrome *c*_553 _from the strictly aerobic hyperthermophilic archaeon, *A. pernix*. SDS-PAGE analysis showed 3 bands at apparent molecular masses of 40, 30, and 25 kDa (Figure 4a, *panel 1*). The measured molecular mass of the 25-kDa band, which was positive for heme staining, was close to the calculated molecular mass for the hypothetical cytochrome *c *subunit encoded by ORF *APE_1719.1 *(Figure 5). Cytochrome *c*_553 _preparations contained heme B and heme C (Figure 2b, *solid line*) and catalyzed electron transfer from menaquinone to yeast cytochrome *c*. On the basis of these results, we concluded that cytochrome *c*_553 _was part of the cytochrome *bc *complex and that the 3 bands identified by SDS-PAGE analysis corresponded to cytochrome *b*, Rieske/FeS, and cytochrome *c *subunits. Data from BN-PAGE analysis supported the idea that these 3 bands are part of the *bc *complex (Figure 4a, *panel 3*). The gene for the cytochrome *c *polypetide, *APE_1719.1 *contains a CXXCHX_n_M motif but does not show high sequence similarity to cytochrome *c*_1 _or the other classes of bacterial or eukaryotic *c*-type components. It is generally difficult to isolate *bc *complexes from membranes because of their general instability, but the heat stability of this enzyme probably permitted its isolation in this study.

We also isolated a cytochrome *oa*_3_-type cytochrome *c *oxidase from *A. pernix *membranes. Based on polypeptide sizes, the upper 2 bands identified by SDS-PAGE (Figure 4b, *panel 1*) probably corresponded to AoxA (subunit I + III) and AoxB (subunit II). Thus, the partially purified cytochrome *oa*_3 _oxidase here is likely the A-type oxidase identified by Ishikawa *et al*. previously [10]. Interestingly, cytochrome *oa*_3 _oxidase comigrated with the *bc *complex through the DEAE-Toyopearl and Q-Sepharose chromatographies, but the enzymes were separated during the subsequent hydroxyapatite chromatography (Figs. S1 and S2). Furthermore, peak fractions from the Q-Sepharose column, which included the *bc *complex and cytochrome *oa*_3 _oxidase, had menaquinol oxidase activity. These findings suggest that cytochrome *oa*_3 _oxidase forms a supercomplex with the *bc *complex as observed in some species, such as thermophilic *Bacillus *PS3 [21], *Corynebacterium glutamicum *[22], and *S. acidocaldarius *[15,23].

## Conclusions

Here, we showed that *A. pernix *has a *bc *complex which includes a *c*-type cytochrome, and that the *bc *complex forms supercomplex with the cytochrome *oa*_3 _oxidase. An electron donor candidate for cytochrome *c *oxidase, such as a blue copper protein, has not yet been identified in the whole genome data of this archaeon. Taken together, it might be suggested that the cytochrome *c*_553 _is the direct electron donor for the oxidase, which would explain the apparent lack of a donor such as a copper protein. We are currently trying to identify an authentic substrate between a *bc *complex and terminal oxidase.

## Methods

### Bacterial strain and growth conditions

*A. pernix *K1 cells were kindly provided by Dr. Yosuke Koga, University of Occupational and Environmental Health, Japan. *A. pernix *was aerobically grown in 5 × T medium [2.8% (w/v) NaCl, 0.067% (w/v) KCl, 0.55% (w/v) MgCl_2_·6H_2_O, 0.69% (w/v) MgSO_4_·7H_2_O, 0.15% (w/v) CaCl_2_, 0.1% (w/v) Na_2_O_3_S·5H_2_O, 0.5% (w/v) Trypticase Peptone, 0.1% (w/v) Yeast Extract, pH 7.0] at 90°C. The preculture was carried out for 48 h in a Sakaguchi-flask containing 50-ml of medium, and a 50-ml aliquot was inoculated into a 1-L culture in a 3-L baffled flask. Cultures were incubated for about 48 h with vigorous shaking (150 rpm) until they attained the early stationary phase of growth. The cells were collected by centrifugation at 5,000 × g for 20 min.

### Membrane preparation

The cells were washed twice with 20 mM NaP_i _buffer at pH 7.0 and re-suspended in the same buffer. The cells were disrupted by sonication with an Ultrasonic Disrupter UD-201 (TOMY, Tokyo) using a 50% duty cycle at output 3 for 20 sec 3 times. The broken cells were precipitated by centrifugation at 16,000 × g for 20 min at 4°C. The precipitate was resuspended in 10 mM Tris-HCl buffer at pH 8.0, which contained a final concentration of 10 mM MgCl_2 _and 10 μg ml^-1 ^DNase, and incubated at 37°C for 30 min. To remove unbroken cells, the suspension was centrifuged at 1,000 × g for 5 min at 4°C. The supernatant was then centrifuged at 100,000 × g for 20 min at 4°C. The precipitate was resuspended in 20 mM NaP_i _at pH 7.0; this suspension was designated as the membrane fraction.

### Solubilization and separation of cytochromes

The membranes were suspended in buffer containing 1 M LiCl and 20 mM NaP_i _at pH 7.0, and then collected by centrifugation. The membrane proteins were solubilized at 10 mg protein ml^-1 ^in 1% (w/v) *n*-dodecyl-β-D-maltoside (DDM) in the presence of 0.3 M NaCl, 20 mM NaP_i _at pH 7.0, and several protease inhibitors [1 mM ethylenediamine-*N*,*N*,*N*',*N*'-tetraacetic acid (EDTA), 0.1 mM phenylmethylsulfonyl fluoride (PMSF), and 0.5 mM benzamidine at final concentrations]. The mixture was centrifuged at 100,000 × g for 30 min, and the supernatant was dialyzed against 10 mM Tris-HCl at pH 7.0.

Cytochromes were separated into 2 components using 3 consecutive chromatography columns: DEAE-Toyopearl, Q-Sepharose, and hydroxyapatite. In brief, the solubilized protein was applied to a DEAE-Toyopearl column after dialysis. The adsorbed proteins were eluted with 3 column volumes of buffer containing 0.1% DDM, 10 mM Tris-HCl at pH 7.0, and an increasing concentration of NaCl (stepwise gradient of 20, 50, 100, 200, 300, and 500 mM). The peak fractions were dialyzed against 10 mM Tris-HCl at pH 7.0 and were applied to a Q-Sepharose column. The proteins were eluted with 15 column volumes of buffer containing 0.1% DDM, 10 mM Tris-HCl at pH 7.0, and an increasing concentration of NaCl (linear gradient of 0-300 mM; Additional file 1). The peak fractions were applied to a hydroxyapatite column for separation. The proteins were eluted with 3 column volumes of buffer containing 0.1% DDM and an increasing concentration of NaP_i _at pH7.0 (stepwise gradient of 20, 50, 100, 150, 200, 300, and 400 mM; Additional file 2).

### Enzyme activities

Cytochrome oxidase activity was assayed at 60°C by measuring oxidation of a yeast cytochrome *c *(Sigma-Aldrich, St. Louis MO), which had been reduced with sodium dithionite, in a final volume 800 μL containing a suitable amount of enzyme, 20 mM NaP_i _at pH 7.0, and 10 μM yeast cytochrome *c*. The oxidation of reduced cytochrome *c *was followed by measuring the decrease in absorbance at 549 nm, and activity was calculated using a millimolar absorption coefficient of 21.2 mM^-1 ^cm^-1 ^[24].

*N*,*N*,*N*',*N*'-Tetramethyl-*p*-phenylenediamine (TMPD) oxidase activity was assayed by measuring the increase in absorbance at 562 nm using a mixture of 25 mM TMPD, 0.1 M NaCl, and 50 mM NaP_i _at pH 6.5, and calculated using a millimolar absorption coefficient of 10.5 mM^-1 ^cm^-1^. To avoid the auto-oxidation of TMPD, the assay was performed at 40°C.

Menaquinol oxidase activity was assayed at 40°C by measuring the oxidation rate of menaquinol-1, which had been reduced with sodium dithionite, in a final volume of 700 μL containing a suitable amount of enzyme, 20 mM NaP_i _at pH 7.0, 0.1% (w/v) DDM, 1 mM EDTA, and 0.2 mM menaquinol-1. The oxidation of reduced menaquinone was followed by measuring the increase in absorbance at 270.7 nm, and the activity was calculated using a millimolar absorption coefficient of 8.13 mM^-1 ^cm^-1^.

### Electrophoretic analyses

Blue-native polyacrylamide gel electrophoresis (BN-PAGE) was performed according to the method of Schägger *et al*. [25]. Nondenaturating electrophoresis was started at 100 V until the sample was within the stacking gel and continued with the voltage and current limited to 350 V and 15 mA, respectively. For two-dimensional analysis, a slice of the BN-PAGE gel was excised and soaked in 1% sodium dodecyl sulfate (SDS) and 1% mercaptoethanol buffer for 1 h and then embedded in a separating gel containing 15% acrylamide. Two-dimensional analysis was performed at room temperature with the current limited to 20 mA. SDS-PAGE was performed according to the method of Laemmli [26]. The gel was stained for protein with CBB and for heme with *o*-toluidine in the presence of H_2_O_2_. Gels were immersed in a solution containing 1% (w/v) o-tolidine, 80% (v/v) CH_3_OH and 10% (v/v) CH_3_COOH for 10 min, and then H_2_O_2 _was added at final concentration of 1% (v/v).

### Mass analysis

Matrix-assisted laser desorption ionization, time-of-flight (MALDI-TOF) mass spectrometry of proteins was performed using 2- (4-hydroxyphenylazo) benzoic acid (HABA) as the matrix as described by Ghaim *et al*. [27]. The cytochromes extracted from the SDS-PAGE gel were precipitated with trichloroacetic acid (TCA) and were dissolved in 99% formic acid before mixing at a 1:5 ratio with a 50% acetonitrile solution containing 1.3 mg HABA ml^-1 ^and 0.1% trifluoroacetic acid. The mixture was spotted onto a sample plate and analyzed using a MALDI-TOF mass spectrometer.

For heme analysis, heme was extracted from partially purified cytochrome *oa*_3 _oxidase with acetone containing 10% concentrated HCl as described previously [28]. After centrifugation, the heme in the supernatant was extracted with ethyl acetate. The heme-containing upper phase was removed, and the ethyl acetate was evaporated under a stream of nitrogen. Heme was dissolved in 30% acetonitrile and then mixed at a 1:1 ratio with a 50% acetonitrile solution containing 10 mg α-cyano-4-hydroxy cinnamic acid ml^-1 ^and 0.1% trifluoroacetic acid. The mixture was spotted onto a sample plate and analyzed using a MALDI-TOF mass spectrometer.

### Additional analyses

Absorption spectra were measured with a recording spectrophotometer (Beckman DU70) at room temperature. Spectra of pyridine ferro-hemochromes were measured in the presence of 10% (v/v) pyridine, 0.05 N NaOH, and 1% (w/v) SDS. For membrane preparations, samples were mixed with 5% (w/v) Triton X-100 and centrifuged at 100,000 × g for 20 min at 4°C, as a common procedure to minimize turbidity. Protein concentration was determined using a modified Lowry method [29].

## Abbreviations

DDM: *n*-dodecyl-*β*-D-maltoside; DCIP: 2,6-dichloroindophenol; menadione, 2-methyl-1,4-naphtoquinone; MALDI: matrix-assisted laser desorption ionization; TOF: time-of-flight; TMPD: *N*,*N*,*N*',*N*'-tetramethyl-*p*-phenylenediamine; BN: blue native; SDS: sodium dodecyl sulfate; PAGE: polyacrylamide gel electrophoresis.

## Authors' contributions

YK carried out the majority of the experimental work, analyzed the data and participated in drafting the manuscript. JS conceived the study, and participated in its design and coordination and helped to draft the manuscript. All authors read and approved the final manuscript.

## Supplementary Material

Additional file 1 **Supplemental Figure S1- Partial purification of cytochrome *bc*-*oa*_3 _supercomplex with Q-Sepharose**. DEAE-Toyopearl chromatography fractions containing both cytochrome *c*_553 _and cytochrome *oa*_3 _oxidases were applied to a Q-Sepharose column for further purification. The cytochrome *c*_553 _eluted together with the cytochrome *oa*_3 _oxidase at ~200 mM NaCl. The peak fraction catalyzed both TMPD oxidation and menaquinol oxidation. Click here for file

Additional file 2 **Supplemental Figure S2- Separation of the cytochrome *bc *complex from cytochrome *bc*-*oa*_3 _supercomplex with hydroxyapatite column chromatography**. Q-Sepharose fractions containing both cytochrome *c*_553 _and cytochrome *oa*_3 _oxidases were applied to a hydroxyapatite column for separation cytohcrome *c*_553 _and cytochrome *oa*_3 _oxidase. The cytochrome *c*_553 _was mainly eluted with 50 mM NaP_i_, and the TMPD oxidase activity was mainly eluted with 300 mM NaP_i_. Click here for file
