# Supplementary material for: Purification and biochemical properties of a cytochrome bc complex from the aerobic hyperthermophilic archaeon Aeropyrum pernix
Source: BMC Microbiol. 2011 Mar 14;11:52. doi: 10.1186/1471-2180-11-52 (PMC3062577; doi:10.1186/1471-2180-11-52)
Supplement: Additional file 1 — Supplemental Figure S1- Partial purification of cytochrome bc-oa3 supercomplex with Q-Sepharose. DEAE-Toyopearl chromatography fractions containing both cytochrome c553 and cytochrome oa3 oxidases were applied to a Q-Sepharose column for further purification. The cytochrome c553 eluted together with the cytochrome oa3 oxidase at ~200 mM NaCl. The peak fraction catalyzed both TMPD oxidation and menaquinol oxidation. [file 1471-2180-11-52-S1.DOC]

**Figure S1**

Q-Sepharose
