# Supplementary material for: Purification and biochemical properties of a cytochrome bc complex from the aerobic hyperthermophilic archaeon Aeropyrum pernix
Source: BMC Microbiol. 2011 Mar 14;11:52. doi: 10.1186/1471-2180-11-52 (PMC3062577; doi:10.1186/1471-2180-11-52)
Supplement: Additional file 2 — Supplemental Figure S2- Separation of the cytochrome bc complex from cytochrome bc-oa3 supercomplex with hydroxyapatite column chromatography. Q-Sepharose fractions containing both cytochrome c553 and cytochrome oa3 oxidases were applied to a hydroxyapatite column for separation cytohcrome c553 and cytochrome oa3 oxidase. The cytochrome c553 was mainly eluted with 50 mM NaPi, and the TMPD oxidase activity was mainly eluted with 300 mM NaPi. [file 1471-2180-11-52-S2.DOC]

**Figure S2**

Hydoroxyapatite
